# The potential of interleukin-37 as an effective therapeutic agent in asthma

**DOI:** 10.1186/s12931-017-0675-x

**Published:** 2017-11-14

**Authors:** Lina Zhang, Jie Zhang, Peng Gao

**Affiliations:** 1 Department of Respiratory Medicine, The Second Hospital of Jilin University, Changchun, Jilin, China; 2 Department of Gastroenterology, Changchun Central Hospital, Changchun, Jilin, China

**Keywords:** Interleukin-37, Asthma, Inflammation, Airway hyper-responsiveness, Leukocytes

## Abstract

Interleukin (IL)-37 belongs to the IL-1 cytokine family. It binds to IL-18Rα and recruits the orphan decoy IL-1R8. Emerging evidence shows that IL-37 is a key player in the regulation of inflammation, cellular differentiation, and proliferation. Altered IL-37 expression has been demonstrated in many inflammatory disease conditions, including asthma. In rheumatoid arthritis, IL-37 is involved in the regulation of proliferation, production of inflammatory mediators, and activation of inflammatory cells. Furthermore, this cytokine acts as a negative regulator of inflammation in inflammatory bowel disease. Similarly, IL-37 also appears to suppress allergic inflammation in asthma. In a murine model of asthma, local administration of IL-37 markedly reduced the degree of inflammatory cell infiltration and airway hyper-responsiveness. IL-37 has also been shown to be involved in a number of aspects of allergic inflammation, such as eosinophil and neutrophil recruitment, as well as inhibition of Th1/Th2/Th17 inflammatory mediators. However, the exact molecular mechanisms underlying the function of IL-37 in human asthma have yet to be fully elucidated. This review describes the current evidence regarding the role of IL-37 in the pathophysiology of asthma and evaluates both the potential of IL-37 as a biomarker for airway inflammation and a therapeutic target for the treatment of asthma.

## Background

**Fig. 1:**
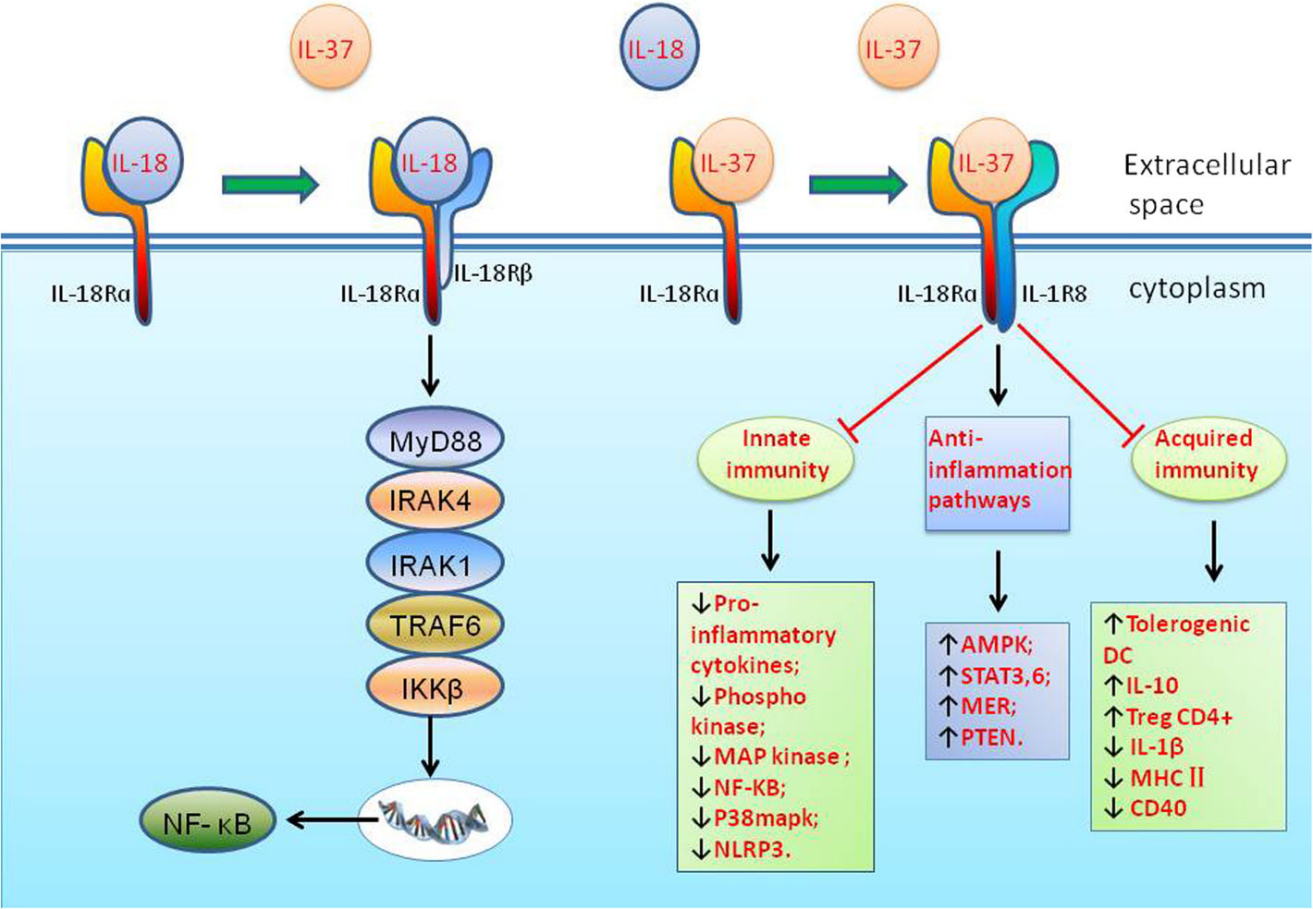
IL-37 binding and signal transduction. IL-37 binds to IL-18Rα instead of IL-18 and recruits the orphan decoy IL-1R8, which leads to suppression of innate and acquired immunity, as well as augmentation of anti-inflammatory pathway, instead of activation of the IL-18 pathway. Adapted from Novick (2013) [61] and Dinarello (2016) [62]

Interleukin (IL)-37 was first described using in silico research in 2000, and increasing evidence has recently demonstrated that IL-37 has profound immunoregulatory functions [1–4]. These include cell proliferation, differentiation, suppression of acquired immunity, and limiting of innate inflammation (Fig. 1). IL-37 is a member of the IL-1 cytokine family that binds to IL-18Rα, which prevents it from binding IL-18 [5]. To date, 11 members have been identified that belong to the IL-1 family. Seven of those components (IL-1α, IL-1β, IL-18, IL-33, IL-36α, IL-36β, and IL-36γ) have pro-inflammatory activities, while there are four antagonists, including IL-1R antagonist, IL-36R antagonist, IL-37, and IL-38 [6]. The only member of the IL-1 family where simian and murine homologues remain undiscovered is IL-37 [3, 7]. In humans, the IL-37 gene is located on chromosome 2 next to genes encoding 8 other members of the IL-1 family. Five splice transcript variants encode distinct isoforms of IL-37 (IL-37a-e) [1–4, 8]. IL-37b is the largest among the isotypes, and has 5 of the 6 exons [9, 10].

In the last decade, research has extensively demonstrated functions of IL-37 in both the innate and adaptive immune system. IL-37 suppresses the production of pro-inflammatory cytokines such as TNF-α, IL-1α, IL-1β, IL-6, G-CSF and GM-CSF from human blood LPS-stimulated monocytes [3, 11]. IL-37 can also reduce neutrophil infiltration into the lung [12] and monocyte infiltration following myocardial infarction [13]. Furthermore, it decreases dendritic cell (DC) activation and has a suppressive action in adaptive immunity [14]. Therefore, the main function of IL-37 is believed to be the reduction of excessive inflammation in both the adaptive and innate immune system through means of negative feedback [15].

IL-37 can exert its anti-inflammatory effects in both intracellular and extracellular conditions. After synthesis, one third of the IL-37b precursor is cleaved intracellularly and then translocated to the nucleus. This process is likely to be associated with Smad3 [3, 16]. On the other hand, cells transfected with IL-37 release an IL-37 precursor after LPS stimulation, which is independent of caspase-1 cleavage at D20 [17]. Extracellular IL-37 executes its anti-inflammatory effects by preventing IL-18Rα from adhering to IL-18 and recruiting the orphan decoy IL-1R8 to the IL-18Rα/IL-37/ IL-1R8 complex (Fig. 1) [11, 18, 19]. Neutralizing antibodies against IL-37 can enhance production of pro-inflammatory cytokines when it is either administered in vitro or in vivo [17, 20].

In recent decades, research activity focusing on IL-37 has increased rapidly. This might be due to the long list of diverse functions demonstrated by IL-37, and its active role in the physiopathology of multiple diseases (Table 1) including rheumatoid arthritis (RA), inflammatory bowel disease (IBD), and asthma. This present review summarizes the published literature on the functions and mechanisms of IL-37 in both humans and experimental models, and explores its role in the pathophysiology of asthma.

**Table 1:** The levels of serum IL-37 in different diseased patients

Disease	Method and assay	Level in blood/tissue fluid (pg/ml)	Comments
Inflammatory Bowel Disease (IBD)	ELISA (Bio-Swamp)	Crohn’s disease: 481.67 ± 232.82(ng/mL); Ulcerative colitis (UC): 199.28 ± 38.60(ng/mL); Healthy control: 2275.68 ± 261.24(ng/mL). Mild UC: 346.97 ± 105.83(ng/mL); Moderate UC: 154.21 ± 24.95(ng/mL); Severe UC: 117.75 ± 17.14(ng/mL).	Serum IL-37 levels are decreased in IBD patients and also differed significantly between mild and moderate/severe UC [63].
Proliferative Diabetic Retinopathy (PDR)	ELISA (R&D Systems)	In vitreous fluids, PDR group: 95.09 ± 5.22 Control group: 34.91 ± 5.61.	The level of IL-37 is elevated in vitreous fluids of patients with PDR compared to controls and correlates with the level of VEGF-A and Ang-2 [64].
Rheumatoid Arthritis (RA)	ELISA (AdipoGen AG, Liestal, Switzerland)	RA patients: 284.7 ± 151.0, Healthy control: 84.64 ± 36.8.	Serum IL-37 levels were increased in patients with RA and were positively associated with disease activity, IL-17/IL-23, and bone loss in RA [65].
Acute ST-Segment Elevation Myocardial Infarction after PCI	ELISA (Adipogen AG, Liestal, Switzerland)	12 h after PCI: 82.8 ± 14.79, 24 h after PCI: 82.2 ± 9.28, 48 h after PCI: 84.4 ± 13.35, Healthy controls: 120.6 ± 2.67.	Plasma and leukocytic IL-37 expression decreased in patients with acute ST-segment elevation myocardial infarction after PCI compared to controls [66].
Arterial Calcification	ELISA (Adipogen AG)	Coronary Artery Calcification (CAC): 72.17 ± 19.74, Non-coronary artery calcification (NCAC): 54.83 ± 18.63. Low-grade CAC: 61.24 ± 15.14, Remarkable CAC: 87.69 ± 14.42.	Increased IL-37 concentrations are associated with the onset of arterial calcification [47].
Systemic Lupus Erythematosus (SLE)	ELISA (R&D Systems Inc)	Normal controls: 10.49 (4.90, 14.94), SLE patients: 13.63 (7.77, 18.24).	Elevated plasma IL-37 levels are seen in SLE patients and are associated with anti-Sm, anti-RNP and C3 [67].
Asthma	ELISA (Adipogen AG)	Asthmatic patients: 48.15 ± 8.49, Healthy controls: 98.59 ± 21.83. Mild asthma: 48.15 ± 8.67, Moderate: 48.30 ± 8.53.	The serum level of IL-37 in asthmatic patients was significantly lower compared to healthy controls. There was no statistically significant difference between the mild and moderate group [42].
Asthma	Real-time quantitative PCR	The relative expression of IL-37 mRNA in asthmatic patients was significantly lower than in healthy controls (*P* = 0.0001).	IL-37 mRNA levels in serum and induced sputum were significantly lower in asthma patients compared to healthy controls. [42].

Data are expressed as mean ± SD or median (IQR)

## IL-37 expression under inflammatory conditions

IL-37 is expressed by a wide range of different tissue and cell types under normal physiological conditions. Cells and tissues that express IL-37 include human blood monocytes, plasma cells, synovial cells, tissue macrophages, tonsillar B cells, epithelial cells (skin and intestine), neoplastic cells, and kidney cells [3]. The level of IL-37 was markedly augmented at 24 h and 72 h in human IL-37-expressing transgenic mice (hIL-37tg mice) after spinal cord injury, while it was barely detectable under normal conditions [21]. The extracellular concentration of IL-37 is aberrantly expressed in a number of inflammatory diseases in humans. Increased levels of IL-37 have been detected in plasma and PBMCs from HIV-1-infected subjects compared to non-infected individuals [22]. Similar results were reported when the serum and synovial fluid of patients with RA was examined, and serum from patients with Graves’ disease also has increased levels of IL-37 [23–25]. In RA patients, IL-37 levels significantly correlated with the rheumatoid factor (RF) value, disease activity, and the levels of IL-4, IL-7, IL-10, IL-12, and IL-13. The plasma level of IL-37 is also decreased in those who respond to treatment with disease-modifying anti-rheumatic drugs (DMARD) [23, 25, 26]. However, the opposing phenomenon is observed in some other diseases. For example, in Behcet’s disease (BD), both mRNA and protein expression of IL-37 was significantly reduced in PBMCs from patients with active disease compared to non-diseased individuals [27]. Moreover, decreased production and expression of IL-37 was also noted in re-stimulated PBMCs from children with allergic bronchial asthma [19, 28].

Both surface expression and intracellular production of IL-37 increase following stimulation with pro-inflammatory cytokines IL-1β, IL-18, TNF-α, and IFN-γ, and anti-inflammatory cytokines TGF-β, IL-10, as well as various toll-like receptor (TLR) ligands [3, 29]. The level of IL-37 in human blood monocytes and DCs rapidly increases under inflammatory conditions, even though it is very low under basal conditions [3, 14, 30]. The inflamed mucosa of IBD patients expressed very high IL-37b levels compared to the normal colonic mucosa evaluated by immunohistochemistry, real time-polymerase chain reaction (PCR), and Western blotting [31]. Similar results were also obtained from the epithelial cells and submucosal lymphoid cells of pediatric patients with chronic IBD [32]. Flow cytometry of circulating B cells, active natural killer cells, and monocytes in patients with IBD showed increased production of IL-37 compared to the control group [33]. Interestingly, endogenous glial cells are likely to be the early source of IL-37 in mice spinal cords, while macrophages are the main IL-37-expressing cells in IL-37tg mice after several types of inflammatory challenge [3, 30, 34]. This is supported by the low level of leukocyte infiltration at the early stages of the immune response instead of endogenous glial cells [35].

Inflammatory states in general are likely to increase the expression of IL-37. For example, IL-37 expression was found to have a non-independent positive association with viral infection [36]. Furthermore, chronically infected HBV patients with high viral loads had higher serum levels of IL-37 compared to non-infected individuals [37]. Environmental stimuli can also affect the production of IL-37. Di Stefano and colleagues reported that smokers had decreased expression of IL-37 in the submucosa compared with healthy non-smokers [38].

## Regulation of leukocyte trafficking and activation

### Macrophage/monocyte

**Fig. 2:**
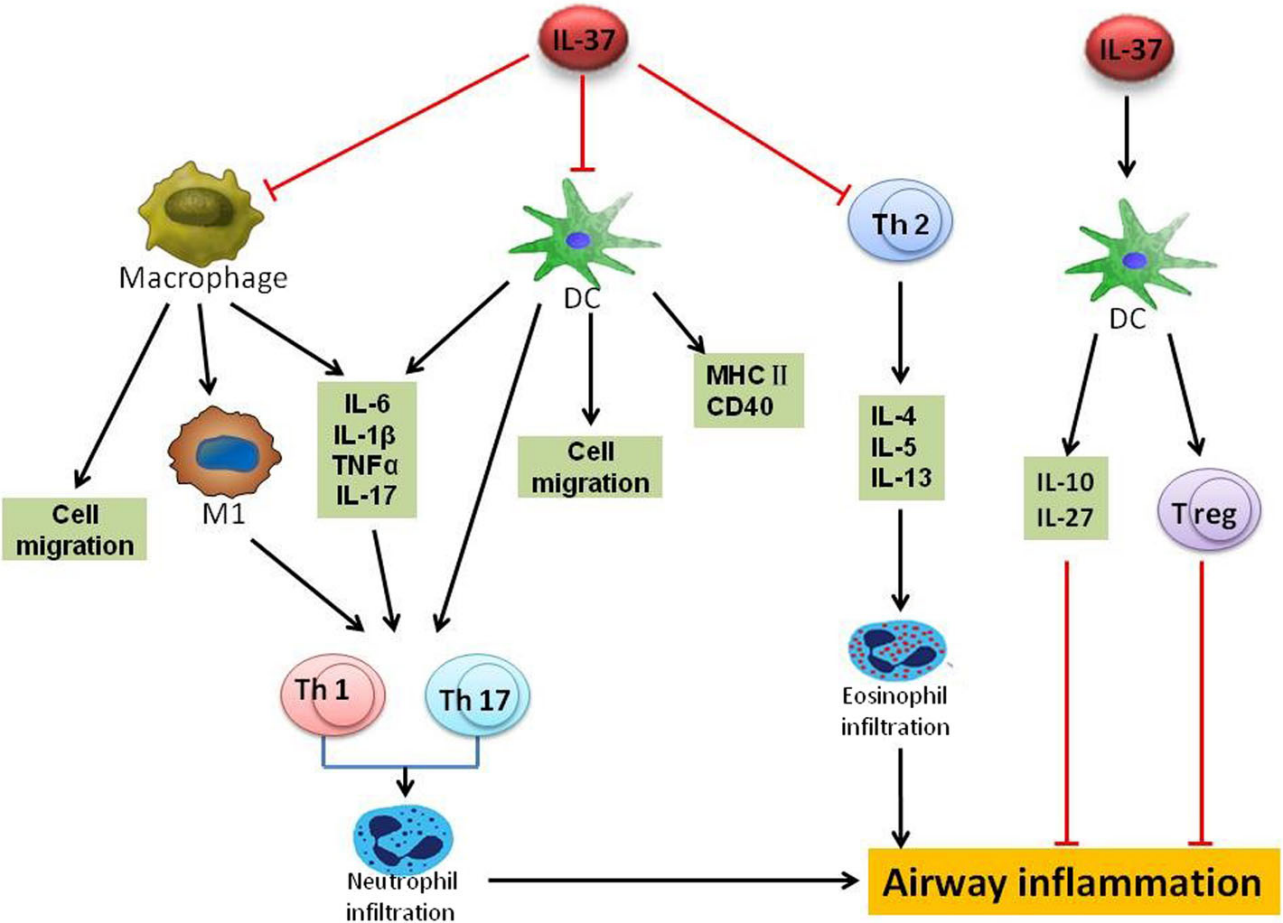
The function of IL-37 in asthma. The black arrow indicates positive effects, while the red T-shaped lines indicate negative effects. CD: Cluster of differentiation; DC, dendritic cell; IL, interleukin; MHC, major histocompatibility complex; Th, helper T cell; TNF, Tumor necrosis factor; T reg, T regulatory cell

As an immune-suppressive factor, IL-37 exerts a vital role in the trafficking of macrophages and monocytes to areas of inflammation. IL-37-expressing transgenic mice exhibited decreased numbers of macrophages and blood-borne neutrophils in comparison to wild-type (WT) mice, along with decreased levels of other cytokines (Fig. 2) [21]. IL-37 can also suppress macrophage production of pro-inflammatory factors. In human blood-derived M1-differentiated macrophages that are highly inflammatory, the rIL-37 precursor decreased LPS-induced IL-6 secretion by approximately 50%. Moreover, LPS-induced secretion of IL-6, TNF-α, and IL-1β are enhanced by administration of a monoclonal anti-IL-37 neutralizing antibody. Mechanistically, IL-37 promotes the increase in IL-1R8 mRNA levels in M1 macrophages, which leads to the dampening of LPS-induced p38 MAPK and pERK, and consequently resulted in anti-inflammatory effects [20]. Similar results were also found in human THP-1 macrophages [39], in those with rheumatoid arthritis [26] and systemic lupus erythematosus [40], in PBMCs of patients with ankylosing spondylitis [41], and in monocytes from human sputum [42]. IL-37 can also modulate macrophage polarity. Huang and colleagues cultured human PBMCs with oxygenated low-density lipoprotein (ox-LDL) to induce M1 macrophages. This process was inhibited by the addition of IL-37 [43].

### Neutrophils

IL-37 can suppress the tissue infiltration of neutrophils. In a murine model of myocardial ischemia/reperfusion (I/R), neutrophil extravasation was significantly dampened after treatment with IL-37, and the cardiac troponin T level and size of infarction area were reduced, indicating ameliorated I/R injury. Cardiac function also improved. Furthermore, in vitro, IL-37 also inhibited neutrophil migration towards the chemokine LIX [13]. Similarly, reduced cellular infiltration following inflammation in IL-37tg mice revealed the importance of a number of proteins in mediating leukocyte recruitment. In mice with aspergillosis, IL-37 reduced neutrophil recruitment. This process was not dependent on the induction of IL-10 but rather depended on dampened activation of the NLRP3 inflammasome. When NLRP3-deficient mice were subjected to Aspergillus spores, neutrophil recruitment in the bronchoalveolar (BAL) fluid and lungs, and IL-1β production were lower than in wild-type mice. Furthermore, this suppression was not altered by IL-37 treatment [12]. Similarly, IL-37 also reduced neutrophil infiltration in a mouse model of arthritis treated with streptococcal cell wall fragments [44], and in mice with spinal cord injury [21].

### Dendritic cells and lymphocytes

IL-37 also regulates the migratory pattern, maturation, and activation of dendritic cells (DC). In a murine model of skin contact hypersensitivity (CHS), bone marrow-derived DCs (BMDCs) isolated from transgenic mice expressing the human IL-37b isoform (IL-37tg) displayed a decreased migratory capacity compared to those from WT mice, although the difference was not statistically significant [14]. IL-37 could also suppress the maturation of DC. LPS-induced MHC II and CD40 expression in BMDCs isolated from IL-37tg mice was reduced by 31 and 52%, respectively, compared to WT mice. DCs isolated from IL-37tg mice also exhibited reduced activation and decreased production of IL-1β, IL-6, and IL-12. However, IL-10 secretion increased compared to DCs from WT mice. Furthermore, DCs from IL-37tg mice were tolerogenic and could readily induce T regulatory (Treg) cell production but had a lower ability to stimulate naive T cells and antigen-specific T cells [14]. Other studies have also demonstrated that IL-37 could reduce maturation of DCs [3, 11].

IL-37 produces similar effects in humans as is evidenced by mice models. Upon stimulation with recombinant (r)IL-37, DCs from patients with Behcet’s disease (BD) have decreased IL-6 and IL-1β expression, and increased IL-27 expression. Furthermore, rIL-37 could markedly reduce the production of reactive oxygen species (ROS) and dampen the activation of all three MAPK pathways (ERK1/2, JNK, and p38) in DCs. Th17 and Th1 cell responses were also inhibited in DCs treated with rIL-37. However, rIL-37 did not affect the DC surface markers CD40, CD86, CD80 and HLA-DR, thus its effects are specific [27].

Recombinant IL-37 significantly reduced Th17 cell frequency and the expression of IL-17 in CD4+ T cells and PBMCs from patients with rheumatoid arthritis. Moreover, IL-37 could markedly suppress Th17 cell proliferation, while only slightly affecting Th17 cell differentiation. Administration of adenovirus encoding human IL-37 (Ad–IL-37) to Th17 cells also remarkably decreased the expression of IL-17 and IL-17-driving cytokines in the synovium and joint cells from mice with collagen-induced arthritis (CIA) [45]. A similar result was also found in CD4+ T cells from induced sputum in patients with asthma [42].

### Other cells

IL-37 not only interacts with inflammatory or immune cells but also with structural cells such as bronchial epithelial cells and smooth muscle cells, which are also key targets in asthma. In sputum-cultured cells and in bronchial epithelial cultured cells, addition of recombinant IL-37 partially suppressed production of the inflammatory protein thymic stromal lymphopoietin (TSLP) by bronchial epithelial cells [46]. Recent research found that vascular smooth muscle cells were the main source of IL-37 in patients with coronary artery calcification (CAC). However, the exact interaction mechanism is yet to be revealed [47].

## IL-37 in experimental models of asthma

In the murine ovalbumin (OVA) model of allergic asthma, IL-37 has been shown to have remarkable anti-inflammatory properties. Eosinophil numbers in BAL and airway tissue are significantly reduced by local administration of IL-37. However, administration of IL-37 did not change the numbers of macrophages, lymphocytes, or neutrophils. Examination of periodic acid Schiff (PAS)-stained airway cross-sections showed that IL-37 markedly reduced goblet cell hyperplasia in the OVA-model. Moreover, methacholine provocation indicated that IL-37 could also reduce airway hyper-reactivity (AHR) in asthmatic mice. Treatment of experimental asthma with intranasal rhIL-37 resulted in significantly decreased production of Th2-type cytokines, such as IL-4, IL-5, and IL-13 as well as pro-inflammatory cytokines such as IL-6. Furthermore, the anti-inflammatory action of IL-37 depended on IL18Ra as well as the orphan receptor SIGIRR/IL-1R8 [19].

## IL-37 in human asthma

Asthma is characterized by chronic airway inflammation caused by abnormal T-cell responses [48]. The inflammation is finely modulated by CD4+ T lymphocytes, including Th1, Th2, and Th17 cells [49]. The infiltration of inflammatory cells and release of inflammatory factors leads to increased bronchial contractions termed AHR and asthmatic symptoms such as wheezing, shortness of breath, and chest tightness [50].

Asthma is a heterogeneous disease, and increased efforts have been made in sub-typing the different phenotypes of asthma. A common method of sub-typing asthma assesses the presence of different inflammatory cells found in induced sputum. Asthma can thereby be classified into four different groups: eosinophilic asthma, neutrophilic asthma, paucigranulocytic asthma, and mixed granulocytic asthma. Each sub-type responds to therapy differentially due to distinctly different immunological mechanisms [51–53]. Crucial to the development of “personalized medicine” is the recognition that asthma is not one homogenous disease but that different sub-types of asthma exist. Classical eosinophilic asthma is the best-characterized sub-type and involves allergen-induced activation of Th2 pathways and release of Th2 cytokines such as IL-4, IL-5, IL-9, and IL-13 [54]. Eosinophilic asthma responds well to corticosteroids, which are the first-line therapy currently used against asthma. Neutrophilic asthma, on the other hand, is driven by the activation of the innate immune system including TLRs and NLRP3 inflammasomes by infections and environmental pollutants [55, 56]. This sub-type of asthma is less well-characterized and notoriously resistant to corticosteroid treatment.

IL-37 plays different roles in the pathogenesis of the different phenotypes of asthma. IL-17, which can be suppressed by IL-37 [45], is involved in both neutrophilic and eosinophilic asthma. IL-17 produced by Th17 cells or macrophages not only promotes the infiltration of neutrophils into the lung but recent evidence also suggests that it can contribute to the development of allergic eosinophilic asthma [57, 58], where it acts synergistically with Th2 cytokines to promote AHR [57, 59, 60].

IL-37 can suppress Th1, Th2, and Th17 effector responses to modulate inflammatory responses (Fig. 2) [19, 45]. Defective IL-37 signaling can lead to both Th2- and Th1-mediated inflammatory diseases [23, 25, 26], including asthma [19].

Despite the sound theoretical possibility, the role of IL-37 in asthma is still not well established. Charrad and colleagues found that IL-37 mRNA expression in serum and induced sputum was significantly lower in asthmatic patients than healthy controls, and that IL-37 mRNA expression was associated with asthma severity [42]. Similar data were reported by Lunding et al., who found significantly lower IL-37 production in human PBMCs from children with allergic asthma compared to healthy controls. Furthermore, treatment of PBMCs with anti-CD3/anti-CD28 decreased IL-37 expression in supernatants from asthmatic samples [19]. These results were confirmed by Raedler and colleagues who also found decreased IL-37 expressions in those with allergic asthma compared to healthy controls [28]. A non-allergic asthma group was also included in this study and patients with non-allergic asthma exhibited increased expression of IL-37 compared to those with allergic asthma [28]. Interestingly, recombinant IL-37 has been shown to partially suppress TSLP production in bronchial epithelial cells from asthmatic patients [46]. These contradictory observations from experimental data might be due to the heterogeneity of asthma and explained by the differences in the sub-types of asthma that were studied. Thus, additional studies with carefully phenotyped asthmatic patients are required to fully characterize the expression of IL-37 in asthmatic patients.

## Conclusion

Despite robust evidence showing that IL-37 is an important regulator of inflammatory cell infiltration, activation, and clearance in experimental models, data on the levels and functions of IL-37 in human asthma is still limited. Recent studies using the OVA model of asthma indicate that IL-37 exerts anti-inflammatory effects in vivo and also suppresses allergen-induced AHR. The lack of consistent data in humans might be due to the heterogeneity of asthma. Thus, additional studies with carefully phenotyped asthmatic patients are required to be able to fully characterize the expression of IL-37 in this disease. Targeting the actions of IL-37 might represent a promising strategy for the development of effective therapeutic agents against asthma.

## Abbreviations

AHR: Airway hyper-reactivity
Ang-2: Angiopoietin
BAL: Bronchoalveolar lavage
BD: Behcet’s disease
BMDC: Bone marrow-derived dendritic cells
CAC: Coronary artery calcification
CHS: Contact hypersensitivity
CIA: Collagen-induced arthritis
COPD: Chronic obstructive pulmonary disease
CXC: Chemokine
DC: Dendritic cell
DMARD: Disease-modifying anti-rheumatic drugs
ERK: Extracellular regulated protein kinases
G-CSF: Granulocyte colony-stimulating factor
GM-CSF: Granulocyte-macrophage colony stimulating factor
HIV: Human immunodeficiency virus
HLA: Human leukocyte antigen
IBD: Inflammatory bowel disease
IFN: Interferon
IL: Interleukin
JNK: c-Jun N-terminal kinase
LIX: Lipopolysaccharide-induced
LPS: Lipopolysaccharide
MAPK: Mitogen-activated protein kinase
MHC: Major histocompatibility complex
NCAC: Non-coronary artery calcification
OVA: Ovalbumin
ox-LDL: Oxygenated low-density lipoprotein
PAS: Periodic acid Schiff
PBMC: Peripheral blood mononuclear cell
PCI: Percutaneous coronary intervention
PCR: Polymerase chain reaction
PDR: Proliferative diabetic retinopathy
Ra: Receptor antagonist
RA: Rheumatoid arthritis
RF: Rheumatoid factor
ROS: Reactive oxygen species
SLE: Systemic lupus erythematosus
Th: Helper T cell
THP: Human acute monocytic leukemia cell line
TLR: Toll-like receptor
TNF: Tumor necrosis factor
Treg: T regulatory cell
UC: Ulcerative colitis
VEGF: Vascular endothelial growth factor
WT: Wild-type
